# Climate-related events, human mobility, and migrant health in Latin America and the Caribbean: evidence gaps and public health implications

**DOI:** 10.3389/fpsyg.2026.1935839

**Published:** 2026-09-04

**Authors:** José Sandoval-Díaz, Camila Navarrete-Valladares, Báltica Cabieses, Consuelo Suazo-Muñoz

**Affiliations:** 1 Centro de Estudios Ñuble, Vicerrectoría de Investigación y Postgrado, Universidad del Bío-Bío, Chillán, Chile; 2 Facultad de Ciencias Jurídicas y Sociales, Universidad Adventista de Chile, Chillán, Chile; 3 Centro de Salud Global Intercultural, Facultad de Medicina Clínica Alemana y Facultad de Psicología, Universidad del Desarrollo, Santiago, Chile

**Keywords:** climate-related events, climate-related mobility, human mobility, migrant health, health psychology, social determinants of health, health inequities, Latin America and the Caribbean

## Abstract

**Objectives:**

To map the available evidence on climate-related events, human mobility, and health outcomes in migrant populations across Latin America and the Caribbean.

**Methods:**

We conducted a structured mini review using an evidence-mapping approach to characterize the available literature. Searches were performed in BIREME/LILACS, PubMed, and Web of Science and were complemented by a manual search of the gray literature. The search, selection, data-charting, and reporting procedures were informed by relevant elements of the PRISMA-ScR guidelines and established methodological guidance for evidence synthesis.

**Results:**

Eight publications were included: six empirical qualitative studies, one personal opinion paper, and one scoping review. None of the six empirical studies compared migrant and local populations, and only one incorporated an intra-group comparison. The populations examined were primarily adults and included climate migrants, economic migrants, and forcibly displaced or forced migrants. The events reported encompassed hurricanes, storms, droughts, floods, and shifts in precipitation patterns, although four of the eight publications addressed climate change in general terms. Health effects were sparsely characterized: four of the eight publications reported acute conditions, whereas chronic conditions were not identified and specific infectious diseases were not reported.

**Discussion:**

The available evidence remains limited, heterogeneous, and poorly disaggregated. Future research should specify climate events, mobility conditions, psychosocial vulnerabilities, and health effects in order to inform equitable, rights-based, and community-oriented public-health responses.

## 1 Introduction

Climate change has emerged as one of the most pressing contemporary challenges for Latin America and the Caribbean (LAC), given the magnitude of its effects on the region's environmental, social, and health conditions. One of its most evident manifestations has been the sustained rise in mean temperature, which in 2024 reached +0.90 °C above the 1991–2020 average. That same year was the warmest on record for LAC and the second warmest for Mexico and South America (United Nations, 2025). Within this context, climate variability has heightened population exposure to extreme heat, severe weather events, and the risk of infectious disease transmission.

Among the most visible expressions of climate intensification in LAC are the increasing frequency of heatwaves, prolonged droughts, and forest fires. These events have expanded across the region, generating substantial effects on ecosystems, livelihoods, and population health, particularly in countries with high social and environmental vulnerability (Hartinger et al., 2025).

These processes also have direct consequences for *human mobility*. In 2022, several LAC countries recorded substantial internal displacement associated with floods, heavy rainfall, and hurricanes, including Brazil, Colombia, and Cuba (International Organization for Migration, 2024). Climate-related mobility may involve temporary or permanent relocation, within or across national borders, in response to sudden or progressive environmental changes linked to climate change (International Organization for Migration, 2019).

Without timely climate action before 2050, an estimated 17 million people could be forced to relocate, increasing migration to other cities by 10% (World Bank Group, 2024).

Nevertheless, climate migration does not exhaust the diversity of forms of human mobility observed in the region. Other modalities include *economic migration*, understood as mobility primarily motivated by the search for employment, income, livelihood security, or improved living conditions (Hidalgo, 2024); *forced displacement*, in which individuals are compelled to relocate due to external factors such as generalized violence, armed conflict, or the systematic violation of human rights (Posada, 2009; Sayas, 2010); *refugee status*, in turn, constitutes a legal protection category applicable to persons outside their country of origin who have a well-founded fear of persecution on the grounds of race, religion, nationality, membership of a particular social group, or political opinion (Posada, 2009). This distinction is relevant for situating with greater precision the specificity of mobility associated with climate events, while acknowledging the coexistence of multiple causes and migratory trajectories.

Climate change also affects multiple dimensions of health and wellbeing. Documented consequences include heat-related illness, respiratory and gastrointestinal conditions, vector-borne diseases, and the aggravation of pre-existing health problems. Although direct attribution remains methodologically complex, climate change intensifies underlying risk conditions, increases pressure on health systems, and disproportionately affects socially vulnerable populations (Bárcena et al., 2020).

Within this context, the health of migrants emerges as a priority field for analysis and intervention. Historically, the issue has been shaped by biomedical and securitarian frameworks that, intertwined with discriminatory practices and discourses, have tended to portray migrant populations as a sanitary risk to host societies. Consequently, responses have prioritized epidemiological surveillance, border-health control, and immunization strategies over a comprehensive understanding of health-illness-care processes. However, this approach falls short of capturing the complexity of migration, as it renders invisible the influence of the social determinants of health, conditions of precarity and exclusion, and the structural inequalities that shape mobility trajectories and effective access to care. From this perspective, it becomes necessary to move toward a rights-based, humanized, and intercultural approach that recognizes migrants as subjects of rights and positions dignity, non-discrimination, health equity, and comprehensive protection as guiding principles for public policies and intervention practices (Burgos Moreno and Parvic Klijn, 2011).

From this perspective, evidence from LAC indicates that migrants face an increased risk of infectious diseases due to multiple factors, including limited access to safe water and food, greater exposure to vectors and pathogens during the migratory journey, and heightened vulnerability to sexual and reproductive health risks (Cortes et al., 2025). This evidence is consistent with findings from low- and middle-income countries, where climate-driven displacement and hazards of natural origin have also been associated with adverse maternal and reproductive health outcomes among women (Afzal et al., 2024). Other threats include sexual violence, interruption of treatment for chronic diseases, exposure to contaminated soils, overcrowding, and inadequate sanitation conditions (Cortes et al., 2025). The main health problems reported among migrants from LAC during stays in camps and transit stations include acute respiratory infections, acute diarrhea, ectoparasitoses, helminthiases, mucocutaneous fungal infections, traumatic injuries, and mental-health problems (Cortes et al., 2025).

Building on this structural and rights-based understanding of migrant health, a health psychology perspective helps to understand how environmental stressors, social inequalities, coping, and access to healthcare shape health and wellbeing throughout migration trajectories. It therefore connects migrant psychosocial experiences with the broader public-health consequences of climate-related mobility (Schwerdtle et al., 2018).

Although Cabieses and Huerta (2024) provide a broad synthesis of the health, psychosocial, and infrastructural effects of climate change on migrant populations in LAC, important gaps remain regarding how specific climate-related events intersect with distinct mobility conditions, population groups, and concrete health outcomes. The limited number of publications identified in this review suggests that these gaps reflect not only the need for more focused syntheses but also the scarcity of primary research addressing these specific intersections. Accordingly, this mini review aims to provide a structured synthesis of the available evidence on climate-related events, human mobility, and migrant health in LAC. A recent Chilean thematic report has similarly highlighted that evidence at the intersection of climate change, human mobility, and health remains limited and emerging, despite growing concern about the disproportionate effects of climate-related hazards on migrant, displaced, and territorially vulnerable populations (Cabieses et al., 2026). Specifically, it seeks to: (i) characterize the countries, document types, study designs, and analytical approaches represented in the literature; (ii) describe the mobility categories, population comparisons, and vulnerable groups included; and (iii) synthesize the climate-related events and health outcomes reported. Unlike previous reviews, this study organizes the evidence according to climate-related event, mobility condition, population comparison, vulnerability, and reported health outcome, thereby identifying specific methodological and substantive gaps relevant to future research and public health policy.

## 2 Methods

This mini review adopted a structured evidence-mapping approach to characterize the extent, methodological features, and substantive gaps of the available literature on climate-related events, human mobility, and migrant health in Latin America and the Caribbean (LAC). The search, selection, and reporting procedures were informed by established guidance for transparent evidence synthesis, including relevant elements of the PRISMA-ScR reporting guidelines (Tricco et al., 2018) and methodological guidance for evidence synthesis (Munn et al., 2018).

### 2.1 Databases

The search strategy was applied to studies published between 2014 and 19 September 2025. The sources consulted included PubMed, Web of Science, and the Virtual Health Library/BIREME, including LILACS, complemented by gray literature searches in Google Scholar. PubMed and Web of Science were selected because of their broad coverage of biomedical and multidisciplinary literature, while BIREME/LILACS was included to ensure adequate representation of Latin American and Caribbean research.

### 2.2 Search strategy

The search combined terms related to climate change and extreme events, health outcomes, migration and displacement, and the 33 LAC countries. The complete search strategy is provided in Supplementary Material 1.

### 2.3 Selection process and eligibility criteria

Empirical studies, evidence syntheses, opinion or conceptual papers, and gray-literature documents published between 2014 and 19 September 2025 were eligible when they addressed the intersection of climate-related events or climate change, human mobility, and migrant health in LAC. Documents published in Spanish, Portuguese, or English were considered. Studies conducted outside LAC were also eligible when they examined migrant populations originating from the region or mobility trajectories substantially involving LAC countries.

Study selection was conducted in two stages. Two reviewers independently screened the titles and abstracts of all retrieved records and subsequently assessed the full texts of potentially eligible documents. Disagreements were initially resolved through discussion between the two reviewers. When consensus could not be reached, two additional reviewers were consulted, and the final inclusion decision was made by consensus.

**Figure 1:**
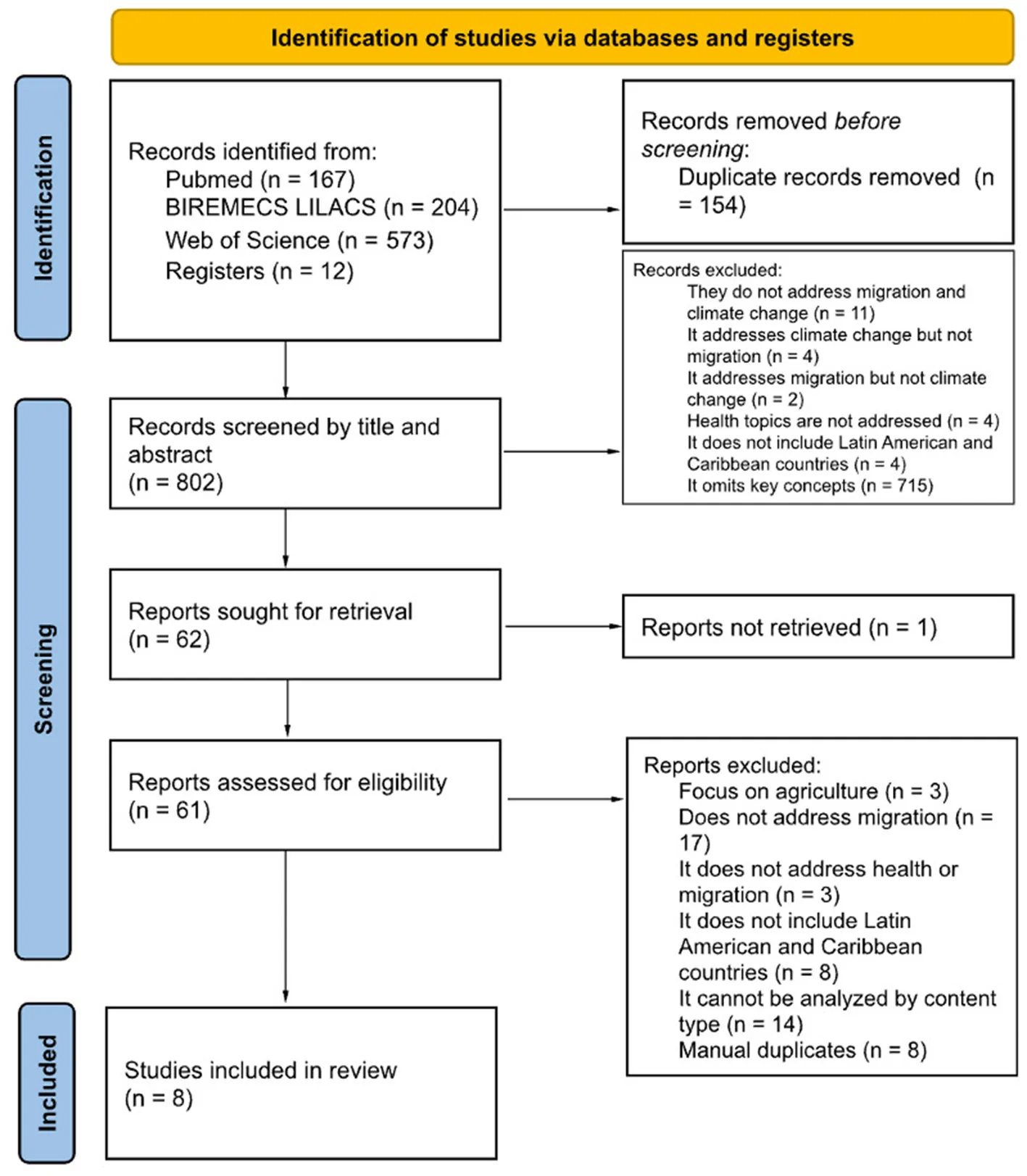
PRISMA flow diagram of the identification, screening, eligibility assessment, and inclusion of studies.

Documents were excluded when they did not address migrant or displaced populations, did not report health-related information, focused exclusively on non-climatic environmental events, or lacked substantive information relevant to the review objectives (see Figure 1). Geophysical events, such as earthquakes, were not classified as climate-related events. However, when they appeared alongside climate-related hazards within an otherwise eligible document, they were retained descriptively and clearly distinguished in the synthesis.

### 2.4 Data extraction and analysis

Data extraction was performed using a charting matrix developed in Excel to systematize relevant information from the included documents. Because this review incorporated multiple types of evidence—including empirical studies, evidence syntheses, conceptual or opinion papers, and gray-literature reports—the charting process was designed to accommodate this methodological diversity. A core set of variables (e.g., authorship, year of publication, geographic focus, climate-related event, migration context, health topic, policy recommendations, and main conclusions) was extracted from all included documents. Methodological variables, including study design, analytical approach, sample characteristics, group comparisons, and specific health outcomes, were extracted only when applicable to empirical studies.

The variables charted, when applicable, were: (i) authorship; (ii) year of publication; (iii) country in which the study was conducted; (iv) country of origin of the migrant population; (v) method; (vi) study design; (vii) type and analytical approach; (viii) type of migration; (ix) inclusion of comparisons between migrant and local populations; (x) presence and description of within-group comparisons; (xi) age group of the sample; (xii) inclusion of vulnerable populations and their characterization; (xiii) type of climate event; (xiv) type of health condition; (xv) classification of conditions as acute or chronic; (xvi) specific health outcomes reported; (xvii) type of infectious disease, when specified; (xviii) incorporation of public-policy recommendations and their description; and (xix) main conclusions.

Given the descriptive purpose of this review, no formal risk-of-bias assessment using standardized appraisal tools was undertaken. Instead, the included publications were characterized using a structured analytical matrix that considered their relevance to the review question, methodological robustness, and primary contribution to the narrative synthesis.

### 2.5 Critical analysis of the evidence

The included publications were characterized using an analytical matrix developed for this review. The matrix considered three dimensions: relevance to the review question, methodological robustness, and primary contribution to the narrative synthesis. This characterization was intended to support the narrative synthesis rather than to provide a formal methodological quality appraisal. The resulting characterization is presented in Table 1.

**Table 1:** Characteristics, methodological robustness, and contribution of the included publications.

References	Country where the study wasconducted	Country of origin of migrants	Methodological approach and analytical focus	Climate or environmental event reported in the study^*^	Type of migrants	Comparison between local and migrant populations	Within-group comparison	Age group of sample	Vulnerable population	Health condition	Relevance to the review question^1^	Methodologicalrobustness^2^	Primary contribution to the synthesis^3^
Batista et al. (2024)	NA	Haiti	Personal opinion on gender, inequality, and human rights	Hurricanes, earthquakes, droughts, rising temperature and precipitation	Climate migrants	NA	NA	—	NA	—	3	2	Conceptual expansion
Cabieses and Huerta (2024)	NA	Mexico, Peru, Honduras, Ecuador, Colombia, Bolivia, Barbuda, Bahamas, Guatemala, Argentina	Scoping literature review	Climate change in general	Climate migrants	NA	NA	—	NA	Acute gas1troin- testinal/ digestive condition; infectious etiology not specified.^**^	5	5	Identification of research gaps
Carreño Calderón et al. (2024)	Chile	Venezuela, Haiti, Colombia, Peru, Bolivia	Qualitative study with thematic analysis focusing on inequality	Climate change in general	Economic migrants	No	No	Adults	Migrants who entered another country irregularly with their children	Acute	5	4	Support for interpretation
Cloos et al. (2023)	Dominican Republic and the island of Guadeloupe	Dominican Republic	Qualitative study with deductive coding focusing on inequality	Hurricanes and storms	Climate migrants	No	No	Adults	Persons experiencing socio-economic vulnerability	Acute	5	4	Empirical evidence
Doering-White et al. (2024)	Mexico	Honduras, Guatemala, Nicaragua	Qualitative study with reflexive thematic analysis	Changes in precipitation	Forcibly displaced/ forced migrants	No	No	Adults and older adults	No	—	5	4	Empirical evidence
Doering-White et al. (2025)	Mexico	Nicaragua, Honduras, Guatemala	Qualitative study focusing on inequality	Drought, floods, irregular rainfall patterns	Forcibly displaced/ forced migrants	No	Comparison of migrants' responses at shelter intake interviews with their responses at in-depth follow-up interviews	Not defined	Migrants forced to leave their countries due to climate events, generalized violence, and food insecurity	Acute	4	3	Applied case
Hasemann Lara et al. (2024)	United States, Mexico, Honduras	Honduras	Qualitative ethnographic study focusing on human rights	Hurricanes and floods	Economic migrants	No	No	Adults	No	—	3	3	Empirical evidence
Watkins et al. (2025)	Chile	Haiti, Venezuela, Dominican Republic, Peru, Bolivia	Qualitative study using discourse analysis and an inductive-interpretative approach focusing on inequality	Climate change in general	No migrant category specified	No	No	Adults	Migrants residing in an informal settlement	Acute	5	4	Support for interpretation

—, information not reported by the publication; NA, not applicable. ^*^Climate and environmental events were recorded as reported in the original publications. Earthquakes were retained as environmental/geophysical events but were not classified as climate-related events. ^**^Gastrointestinal/digestive conditions were differentiated from specific infectious diseases; when an infectious etiology was not explicitly reported, the condition was not classified as an infectious disease. ^1^ Relevance to the review question: 1, very limited; 2, limited; 3, moderate; 4, high; 5, very high. ^2^ Methodological robustness: 1, very low; 2, low; 3, moderate; 4, high; 5, very high. ^3^ Primary contribution to the synthesis reflects the principal role of each publication in supporting the narrative synthesis: background/context, empirical evidence, applied case, support for interpretation, conceptual expansion, or identification of research gaps.

## 3 Results

Table 1 summarizes the characteristics, methodological robustness, and primary contribution of the included publications. The final corpus comprised 8 documents: 6 empirical qualitative studies, 1 personal opinion paper, and 1 scoping review. The analytical approaches used in the empirical studies were heterogeneous: 1 study used thematic analysis, 1 used reflexive thematic analysis, 1 applied deductive coding, 1 employed discourse analysis, 1 adopted an inductive-interpretative approach, and 1 did not explicitly report its analytical approach. Across the 8 publications, 5 examined inequality as a central analytical perspective, 2 addressed human rights, and 1 focused primarily on gender.

Among the 8 included publications, 2 were conducted in Chile and 2 in Mexico. Regarding the countries of origin of the migrant populations, 4 publications included migrants from Honduras, 3 from Haiti, 3 from Peru, 3 from Bolivia, and 2 from Venezuela. The available evidence was geographically uneven and concentrated in a limited number of national contexts.

Regarding mobility categories, 3 of the 8 publications focused on climate migrants, 2 on economic migrants, 2 on forcibly displaced or forced migrant populations, and 1 did not explicitly classify the migrant category. None of the 6 empirical studies compared migrant and local populations, and only 1 incorporated an intra-group comparison by contrasting responses obtained during shelter-intake interviews with those gathered in subsequent in-depth follow-up interviews.

Regarding sample age, 4 of the 6 empirical studies included adults, 1 included adults and older adults, and 1 did not define the age group. Sample age was not applicable to the personal opinion paper or the scoping review. In addition, 4 of the 8 publications examined populations in situations of vulnerability associated mainly with socioeconomic precarity, irregular migration with children, forced displacement linked to climate-related events, violence, food insecurity, and residence in informal settlements.

Across the 8 publications, 3 reported hurricanes, 2 reported droughts, 2 reported floods, 2 reported changes in precipitation or rainfall patterns, 1 reported storms, 1 reported rising temperatures, and 1 reported increased precipitation. One publication also reported earthquakes as an environmental or geophysical event, although earthquakes are not classified as climate-related events. Four of the 8 publications referred to climate change in general terms rather than identifying a specific climate-related event.

Regarding health conditions, 4 of the 8 publications reported acute conditions, whereas the remaining 4 did not specify whether the conditions were acute or chronic. Only 1 of the 8 publications identified gastrointestinal or digestive conditions. None reported chronic conditions or specified a particular infectious disease. Overall, the publications provided limited detail on the links between climate-related events, mobility conditions, and health outcomes, as well as on psychosocial vulnerabilities and barriers to healthcare.

## 4 Discussion

This mini review mapped and synthesized the available evidence on climate-related events, human mobility, and health outcomes among migrant populations in LAC. Given the emerging and heterogeneous nature of this field, the review focused on identifying the extent, characteristics, and principal gaps of the available evidence rather than estimating causal effects, evaluating intervention effectiveness, or conducting a quantitative synthesis (Tricco et al., 2018; Munn et al., 2018).

The findings reveal three main gaps: (i) the predominance of qualitative evidence; (ii) the absence of systematic between-group comparisons; and (iii) the low specification of health effects associated with concrete climate events. These findings suggest that, although the linkage between climate change, human mobility, and health constitutes a growing concern for global public health (McMichael, 2023), the regional evidence remains fragmentary, scarcely comparable, and poorly disaggregated. These gaps also limit understanding of the psychosocial processes that may shape health outcomes among migrant populations exposed to climate-related events (Schwerdtle et al., 2018).

First, the six empirical studies used qualitative approaches to examine migration experiences in contexts of vulnerability. This approach is well suited to an emerging field, as it enables exploration of how climate events articulate with the social, economic, and political conditions that shape human mobility and health-illness-care processes. This reading aligns with the broader literature on environmental migration, which warns that climate-related mobility does not arise from a single factor but from the interplay among environmental conditions, livelihoods, structural inequalities, and adaptive capacities (Black et al., 2011). Nevertheless, the scarcity of quantitative or mixed-methods studies limits the possibility of estimating the magnitude, frequency, and distribution of the reported health effects. Consequently, complementing the interpretive depth of qualitative studies with designs allowing greater comparability and utility for health planning is warranted.

Second, the review reveals a limited use of comparative analyses. None of the six empirical studies systematically compared health effects between migrant and local populations, and only one incorporated an intra-group comparison across distinct data-collection moments. This limitation is significant, given that the literature has indicated that climate-related migration may intensify health risks when combined with environmental exposure, barriers to accessing health services, socio-economic precarity, and limited institutional protection (Schwerdtle et al., 2018). Consequently, the absence of comparative designs constrains the ability to determine with precision whether the reported health effects are differentially distributed across groups, or whether specific migration conditions, such as forced displacement, irregular migration, or residence in informal settlements, amplify these risks.

Third, the studies reviewed address a diversity of climate and hydrometeorological events, including hurricanes, storms, droughts, floods, and changes in precipitation patterns. These phenomena have been recognized as factors that may influence human mobility, either as direct triggers of displacement or as conditions that deepen pre-existing socio-economic vulnerabilities (Black et al., 2011; International Organization for Migration, 2021). Several studies, however, refer to climate change in general terms, without clearly distinguishing the type of event, the duration of exposure, the stage of the migratory trajectory, or the mechanisms through which these events relate to health outcomes. This low level of specification reduces the comparative and analytical capacity of the evidence and reinforces the need to differentiate among climate hazards, hydrometeorological events, and other environmental or geophysical events reported in the literature.

One of the principal gaps identified concerns the limited characterization of health effects. Although 4 of the 8 publications reported acute conditions, the publications generally provided limited detail on the specific health problems observed. No chronic conditions or specific infectious diseases were reported, although 1 publication mentioned gastrointestinal or digestive conditions without specifying an infectious etiology. This lack of disaggregation makes it difficult to distinguish between effects potentially associated directly with environmental exposure and indirect effects linked to the migratory journey, overcrowding, food insecurity, inadequate sanitation, or barriers to care. This finding is consistent with previous reviews characterizing the evidence on climate migration and health as fragmentary and heterogeneous (Mazhin et al., 2020), although risks such as infectious diseases, gastrointestinal disorders, heat stress, dehydration, vector-borne diseases, and mental-health problems have been documented in contexts of climate change and human mobility (Romanello et al., 2021). This limited characterization also restricts understanding of psychosocial adaptation, perceived vulnerability, coping processes, and other psychosocial dimensions relevant to migrant health. These findings allow the contribution of the present review to be situated relative to prior work. While (Cabieses and Huerta 2024) provide a broad overview of the relationship between climate change, migration, and health among migrants in Latin America and the Caribbean, the present review identifies limited systematization when specific climate events, types of human mobility, and differentiated health effects are analyzed jointly. In particular, gaps persist concerning the distinction between acute and chronic conditions, the identification of infectious diseases, comparisons between migrant and local populations, and the characterization of vulnerable groups.

From a public-health perspective, the reported health effects of climate change and human mobility should be understood in relation to the social and environmental determinants of health. Climate events do not act in isolation; rather, they interact with socio-economic, institutional, and territorial conditions that influence the exposure, vulnerability, and health-service access of migrant populations. Thus, migration may operate both as a consequence of exposure to climate risks and as an adaptive strategy; however, when it occurs under conditions of social exclusion, precarity, or weak institutional protection, health risks may be magnified (McMichael, 2023).

The findings of this review carry implications for the formulation of public-health policies attentive to human mobility in the context of climate change. First, epidemiological surveillance and health-information systems must be strengthened by incorporating more precise records of migratory status, type of climate event, exposure conditions, and health effects. This is especially relevant given that migrants may face risks associated with limited access to potable water and food, exposure to vectors and pathogens, overcrowding, inadequate sanitation, and the interruption of chronic-disease treatments (Cortes et al., 2025). Second, equitable access to health services must be guaranteed regardless of migratory status, drawing on a framework of rights, dignity, non-discrimination, and interculturality (Burgos Moreno and Parvic Klijn, 2011). Finally, intersectoral responses are needed that articulate health, migration, housing, sanitation, social protection, and climate adaptation, recognizing that health risks are configured at the intersection of environmental exposure, social inequality, and institutional barriers (McMichael, 2023; Schwerdtle et al., 2018).

In terms of future research, the gaps identified suggest a need for studies that more precisely specify the type of climate event, mobility condition, and reported health effects. In particular, future work should differentiate among acute, chronic, infectious, and psychosocial conditions, and describe barriers to health-service access, given that these dimensions were sparsely characterized in the studies reviewed. Likewise, the absence of comparisons between migrant and local populations, and the limited comparison among migrant subgroups, reinforce the need for comparative designs capable of analyzing differences by age, gender, migratory status, country of origin, stage of the journey, and conditions of settlement. International evidence from gender-specific populations affected by migration also suggests that subgroup-specific analyses can identify distinct determinants of healthcare utilization and access barriers that may remain obscured in more aggregated approaches (Afzal and Das, 2023). Future research should also strengthen interdisciplinary approaches integrating public health, health psychology, migration studies, and climate sciences to better understand the complex pathways linking climate-related mobility and migrant health.

This review has several limitations, including the small and methodologically heterogeneous corpus and the limited specification of health outcomes. Combining empirical studies with a scoping review and an opinion paper reduced comparability and limited the strength of the public-health recommendations. Nevertheless, it provides a structured overview of the evidence on climate-related mobility and migrant health in Latin America and the Caribbean, identifying key methodological and substantive gaps that should guide future interdisciplinary research and the development of more equitable, climate-responsive health policies.
