# Supplementary material for: Climate-related events, human mobility, and migrant health in Latin America and the Caribbean: evidence gaps and public health implications
Source: Front Psychol. 2026 Sep 4;17:1935839. doi: 10.3389/fpsyg.2026.1935839 (PMC13602859; doi:10.3389/fpsyg.2026.1935839)
Supplement: Supplementary file 1 [file Supplementary_file_1.pdf]

Supplementary Material 1

Complete Electronic Database Search Strategies

Climate-related events, human mobility, and migrant health in Latin America and the Caribbean: evidence gaps and public health implications

José Sandoval-Díaz, Camila Navarrete-Valladares, Báltica Cabieses, and Consuelo Suazo-Muñoz

Search strategy

The electronic database search was conducted on 19 September 2025 in PubMed, Web of Science, and the Virtual Health Library (BIREME/LILACS). The search strategy combined four conceptual domains: (1) climate change and climate-related events, (2) health, (3) migration and human mobility, and (4) Latin America and the Caribbean. The complete search strategies and the number of records retrieved from each database are reported below.

Table S1. Summary of electronic database searches

Database	Search date	Combined search	Results, 2014 onwards
PubMed	19 September 2025	176	161
Web of Science	19 September 2025	712	571
BIREME/LILACS	19 September 2025	315	205
Total		1,203	937

Note. The figures reported in this table reproduce the available original electronic database search records. These searches yielded 937 records after application of the documented publication-year limits (PubMed, n = 161; Web of Science, n = 571; BIREME/LILACS, n = 205). The identification process reported in the PRISMA flow diagram additionally incorporated records identified through supplementary manual and gray-literature searching. Therefore, the figures in Table S1 should be interpreted as the documented electronic database search records rather than as a reconstruction of the complete PRISMA identification count.

PubMed

Search date: 19 September 2025

Table S2. PubMed search strategy

Set	Search terms	Results
#1	("climate change" OR "climatic change" OR "global warming" OR "extreme weather" OR "extreme climate" OR "climate variability")	118,286
#2	("health" OR "public health" OR "population health" OR "epidemiology" OR "disease" OR "mortality" OR "death" OR "morbidity" OR "hospitalization" OR "injurie*" OR "illness*" OR "infection" OR "malaise")	15,305,109
#3	("Transients and Migrants"[Mesh] OR "Emigrants and immigrants"[Mesh] OR "Refugee" OR "Migration background" OR "Immigrant background" OR "Migrant" OR "Migrants" OR "Immigrant" OR "Immigrants" OR "migration" OR "displacement" OR "forced migration" OR "environmental migration" OR "climate migration" OR "climate displacement" OR "environmental displacement" OR "population mobility" OR "human mobility" OR "relocation" OR "resettlement" OR "evacuation" OR "climate refugee*" OR "environmental refugee*" OR "internal displacement" OR "relocation")	628,493
#4	("Latin America" OR "South America" OR "Central America" OR "Antigua and Barbuda" OR "Argentina" OR "Bahamas" OR "Barbados" OR "Belize" OR "Bolivia" OR "Brazil" OR "Chile" OR "Colombia" OR "Costa Rica" OR "Cuba" OR "Dominica" OR "Dominican Republic" OR "Ecuador" OR "El Salvador" OR "Grenada" OR "Guatemala" OR "Guyana" OR "Haiti" OR "Honduras" OR "Jamaica" OR "Mexico" OR "Nicaragua" OR "Panama" OR "Paraguay" OR "Peru" OR "Saint Kitts and Nevis" OR "Saint Lucia" OR "Saint Vincent and the Grenadines" OR "Suriname" OR "Trinidad and Tobago" OR "Uruguay" OR "Venezuela")	1,208,893
#5	#1 AND #2	36,249
#6	#1 AND #2 AND #3	1,454
#7	#1 AND #2 AND #3 AND #4	176
Limit	2014 onwards	161

Web of Science

Search date: 19 September 2025

Table S3. Web of Science search strategy

Set	Search terms	Results
#1	("climate change" OR "climatic change" OR "global warming" OR "extreme weather" OR "extreme climate" OR "climate variability")	750,795
#2	("health" OR "public health" OR "population health" OR "epidemiology" OR "disease" OR "mortality" OR "death" OR "morbidity" OR "hospitalization" OR "injurie*" OR "illness*" OR "infection" OR "malaise")	19,587,501
#3	("Transients and Migrants"[Mesh] OR "Emigrants and immigrants"[Mesh] OR "Refugee" OR "Migration background" OR "Immigrant background" OR "Migrant" OR "Migrants" OR "Immigrant" OR "Immigrants" OR "migration" OR "displacement" OR "forced migration" OR "environmental migration" OR "climate migration" OR "climate displacement" OR "environmental displacement" OR "population mobility" OR "human mobility" OR "relocation" OR "resettlement" OR "evacuation" OR "climate refugee*" OR "environmental refugee*" OR "internal displacement" OR "relocation")	1,852,662
#4	("Latin America" OR "South America" OR "Central America" OR "Antigua and Barbuda" OR "Argentina" OR "Bahamas" OR "Barbados" OR "Belize" OR "Bolivia" OR "Brazil" OR "Chile" OR "Colombia" OR "Costa Rica" OR "Cuba" OR "Dominica" OR "Dominican Republic" OR "Ecuador" OR "El Salvador" OR "Grenada" OR "Guatemala" OR "Guyana" OR "Haiti" OR "Honduras" OR "Jamaica" OR "Mexico" OR "Nicaragua" OR "Panama" OR "Paraguay" OR "Peru" OR "Saint Kitts and Nevis" OR "Saint Lucia" OR "Saint Vincent and the Grenadines" OR "Suriname" OR "Trinidad and Tobago" OR "Uruguay" OR "Venezuela")	1,784,872
#5	#1 AND #2	137,621
#6	#1 AND #2 AND #3	6,645
#7	#1 AND #2 AND #3 AND #4	712
Limit	2014 onwards	571

Note. The search terms reproduce the syntax recorded in the original Web of Science search record.

Virtual Health Library (BIREME/LILACS)

Search date: 19 September 2025

Table S4. BIREME/LILACS search strategy

Set	Search terms	Results
#1	("climate change" OR "climatic change" OR "global warming" OR "extreme weather" OR "extreme climate" OR "climate variability")	111,463
#2	("health" OR "public health" OR "population health" OR "epidemiology" OR "disease" OR "mortality" OR "death" OR "morbidity" OR "hospitalization" OR "injurie*" OR "illness*" OR "infection" OR "malaise")	20,290,075
#3	("Transients and Migrants" OR "Emigrants and immigrants" OR "Refugee" OR "Migration background" OR "Immigrant background" OR "Migrant" OR "Migrants" OR "Immigrant" OR "Immigrants" OR "migration" OR "displacement" OR "forced migration" OR "environmental migration" OR "climate migration" OR "climate displacement" OR "environmental displacement" OR "population mobility" OR "human mobility" OR "relocation" OR "resettlement" OR "evacuation" OR "climate refugee*" OR "environmental refugee*" OR "internal displacement" OR "relocation")	947,300
#4	("Latin America" OR "South America" OR "Central America" OR "Antigua and Barbuda" OR "Argentina" OR "Bahamas" OR "Barbados" OR "Belize" OR "Bolivia" OR "Brazil" OR "Chile" OR "Colombia" OR "Costa Rica" OR "Cuba" OR "Dominica" OR "Dominican Republic" OR "Ecuador" OR "El Salvador" OR "Grenada" OR "Guatemala" OR "Guyana" OR "Haiti" OR "Honduras" OR "Jamaica" OR "Mexico" OR "Nicaragua" OR "Panama" OR "Paraguay" OR "Peru" OR "Saint Kitts and Nevis" OR "Saint Lucia" OR "Saint Vincent and the Grenadines" OR "Suriname" OR "Trinidad and Tobago" OR "Uruguay" OR "Venezuela")	974,557
#5	#1 AND #2	40,390
#6	#1 AND #2 AND #3	3,503
#7	#1 AND #2 AND #3 AND #4	315
Limit	2014 onwards	205

SUPPLEMENTARY SEARCH: In addition to the electronic database searches, a supplementary manual search of gray literature was conducted, including Google Scholar, to identify potentially relevant publications not retrieved through the database searches. Records identified through this supplementary search were incorporated into the screening process and are reported separately in the PRISMA flow diagram.
